# Body image flexibility and embodiment in eating disorders: a mixed-methods approach combining network analysis and pilot exposure protocol

**DOI:** 10.1186/s40337-025-01249-3

**Published:** 2025-04-10

**Authors:** Paolo Meneguzzo, Chiara Cazzola, Francesca Buscaglia, Anna Pillan, Filippo Pettenuzzo, Patrizia Todisco

**Affiliations:** 1 Department of Neuroscience, University of Padova, Via Giustiniani 2, Padova, 35128 Italy; 2 Padova Neuroscience Center, University of Padova, Padova, Italy; 3 Eating Disorder Unit, Casa di Cura “Villa Margherita”– KOS Group, Arcugnano (Vicenza), Italy; 4 Vicenza Eating Disorders Center, Mental Health Department, Azienda ULSS8 “Berica”, Vicenza, Italy

**Keywords:** Body image flexibility, Embodiment, Eating disorders, Body image disturbances, Interoception, Self-compassion, Network analysis

## Abstract

**Objective:**

Body image (BI) disturbances are central to the psychopathology of eating disorders (EDs), where body dissatisfaction and disembodiment often co-occur, exacerbating negative body image experiences. We aimed to examine body image flexibility and embodiment in women with EDs and a general population (GP) sample.

**Method:**

Data were collected from 172 participants, including 103 GP women and 69 women with EDs. Standardized questionnaires measuring body uneasiness, interoceptive awareness, and body image acceptance were administered. Additionally, a pilot group therapy intervention targeting body image concerns was evaluated with 24 ED participants.

**Results:**

Findings suggest that significant differences in embodiment-related features emerged (self-regulation and body trust), while both groups reported similar levels of interoceptive awareness. Network analysis revealed distinct patterns of partial correlations between variables within each group, with higher centrality for body image concerns and weight phobia in EDs. In contrast, the GP group exhibited stronger connections between embodiment features such as attention regulation and body image acceptance. The pilot intervention showed promise in improving body image flexibility and reducing body image concerns.

**Conclusions:**

These results underscore the importance of fostering body image flexibility and embodiment in the clinical treatment of EDs, suggesting potential pathways for enhancing therapeutic interventions.

## Abstract (plain language summary)

People with eating disorders often struggle with how they see and feel about their bodies. This study looked at body image flexibility (the ability to accept and adapt to thoughts about one’s body) and body awareness (how well someone can notice and understand their internal bodily sensations). We compared people with eating disorders to those without, examining how these factors were linked. We found that people with eating disorders were more focused on their weight and had greater body image concerns, while those without eating disorders had a stronger connection to their bodily sensations. A small therapy group for women with eating disorders showed that exercises focusing on body awareness and acceptance helped some participants feel more at ease with their bodies. However, some found it emotionally challenging, showing that these approaches need to be adapted to each individual. Our findings suggest that improving body awareness and flexibility could be an important part of eating disorder treatment. Helping people develop a more accepting relationship with their bodies may reduce distress and improve recovery.

## Highlights

• Body image flexibility differs in eating disorders vs. general population groups.

• Network analysis reveals weight phobia’s centrality in eating disorder networks.

• Pilot group therapy improves body trust, flexibility, and reduces weight concerns.

• Exposure-based therapy might address body image distress with mindful techniques.

• Findings support tailored interventions for enhanced body image and resilience in Eds.

## Introduction

Body image (BI) is a multifaceted construct that reflects an individual’s perceptions, attitudes, and beliefs about their body, encompassing cognitive, affective, and behavioral responses [1]. The cognitive component includes thoughts and evaluations about one’s body, such as overvaluation of shape and weight, while the affective component involves emotions and feelings related to body image, including dissatisfaction, anxiety, or shame [2–4]. The behavioral component, including body checking and avoidance behaviors, plays a central role in the maintenance of body image disturbances (BID) [5]. Individuals with BID often engage in repetitive behaviors such as mirror checking or avoidance strategies (e.g., wearing loose clothing), which reinforce negative body-related beliefs and emotional distress [6]. These responses are influenced by a dynamic interplay between individual characteristics—such as body shape, self-esteem, and personality traits—and external forces, including societal beauty standards and media representations [7–9]. While historically approached as a binary construct (positive vs. negative), contemporary perspectives underscore the need to recognize positive and negative BI as coexisting dimensions that shape body-related experiences in nuanced ways [10, 11]. Recent work in this field points to the significance of embodiment, a process through which individuals experience their bodies as integrated aspects of their self-identity [12, 13]. Despite growing recognition of embodiment, limited research has examined how interventions targeting body image flexibility and embodiment may influence BID in individuals with eating disorders (EDs) [14–16].

Embodiment, as outlined in the Developmental Theory of Embodiment [17, 18], suggests that fostering a positive, accepting relationship with one’s body can buffer against BID. In contrast, disembodiment—characterized by detachment, alienation, or shame related to the body—can exacerbate negative body image and is frequently observed in individuals with EDs, where body-related anxiety and avoidance are prevalent [17, 19]. This approach underscores the importance of addressing both embodiment and disembodiment as distinct constructs in body image research, which could lead to more effective, holistic interventions.

Body image flexibility (BIF) emerges as a relevant concept within this context, capturing an individual’s capacity to experience body-related thoughts and emotions with acceptance and non-judgment, even when those experiences are distressing. This flexibility aligns closely with acceptance-based therapeutic models, particularly Acceptance and Commitment Therapy (ACT), which emphasize resilience through acceptance rather than avoidance of body-related distress [20]. BIF has been shown to mitigate negative BI by enabling individuals to engage with negative body thoughts without allowing these to dominate their self-perception or dictate behavior, ultimately fostering a more adaptive, self-compassionate outlook [21, 22].

In the clinical assessment and treatment of BID, particularly among ED populations, positive embodiment and self-compassion play pivotal roles as protective factors [23, 24]. Recent narrative reviews highlight the need to expand the theoretical understanding of BI by incorporating positive embodiment and addressing the ways that self-compassion—defined as a nurturing and non-critical stance toward oneself—can offset body shame and promote a positive, grounded body experience [19, 25, 26]. These factors are critical not only for reducing disembodiment and body shame, which are linked to heightened vulnerability to BID, but also for enhancing BIF, which fosters resilience in the face of body dissatisfaction [27, 28].

Furthermore, to advance our understanding of BID in EDs, it is essential to explore protective factors such as self-compassion and positive embodiment across diverse populations and life stages [29]. Current research has predominantly focused on young, white, cisgender females, limiting the generalizability of findings across different demographic groups and developmental stages [30]. Examining these protective factors in varied populations would provide a more inclusive understanding of BID in EDs. Additionally, emerging literature highlights differences within ED populations based on the duration of the disorder. Findings suggest that the longer an individual has an ED, the more ingrained certain body image disturbances may become, potentially reflecting a ‘habit’ in enduring these disorders [31, 32]. These insights underscore the importance of exploring BID across varying illness durations, as different phases of an ED may influence the individual’s relationship with their body and the effectiveness of protective factors like self-compassion and embodiment.

Although several studies have examined aspects of embodiment in EDs, research on interventions specifically targeting body image flexibility remains scarce. Understanding how different body-related constructs interact and whether targeted interventions can improve BI-related outcomes is crucial. Therefore, this study aims to address these gaps by first exploring the interconnections between body image flexibility, embodiment, and body image disturbances in individuals with eating disorders and the general population. As this part of the study is exploratory, we do not propose specific hypotheses but expect to identify distinct patterns in the relationships between these constructs across clinical and non-clinical groups. Additionally, we conduct a pilot evaluation of a body image intervention in individuals with eating disorders, hypothesizing that participation will lead to increased body image flexibility and body trust, along with reductions in weight phobia and compulsive self-monitoring.

## Methods

### Study design and participants

This study employed a mixed-methods approach, integrating cross-sectional network analysis with a pilot intervention.We included 103 women from the general population and 69 women diagnosed with eating disorders, recruited from Villa Margherita-KOS Group in Arcugnano, Vicenza, Italy. The inclusion criteria for both groups were: age over 16 years, no cognitive impairment, and no diagnosis of a psychotic disorder. For the general population group, an additional criterion was the absence of a lifetime diagnosis of any eating disorder.

In the clinical sample, diagnoses were made by a trained psychiatrist using a semi-structured clinical interview based on DSM-5 diagnostic criteria.

The pilot longitudinal evaluation was performed on 24 women with an ED diagnosis, who performed the target group therapy during their inpatient treatment. These participants were part of a larger sample that completed the questionnaires.

All participants provided written informed consent during recruitment. For participants under 18 years old, consent was obtained from their parents. The study adhered to the ethical standards set forth in the Declaration of Helsinki and was approved by the local ethics committee as part of the clinical evaluation of the patients.

### Material

All participants completed a demographic questionnaire that included information such as age, weight, and height, used to calculate the body mass index (BMI). Additionally, participants filled out three standardized questionnaires: the Body Uneasiness Test (BUT), the Multidimensional Assessment of Interoceptive Awareness (MAIA), and the Body Image Acceptance and Action Questionnaire (BI-AAQ). Participants enrolled in the pilot group exposure treatment were also asked to complete the questionnaires after the group sessions concluded.

The BUT is a self-report questionnaire designed to assess body image disturbances and related psychological constructs [33]. It consists of multiple items that evaluate various dimensions of body uneasiness, including concerns about body shape, weight, and specific bodily sensations. Higher scores on the BUT indicate greater levels of body uneasiness. All the subscales demonstrated good internal reliability in this study, with Cronbach’s alphas > 0.70.

The MAIA is a questionnaire that measures interoceptive awareness, which refers to the ability to perceive and interpret internal bodily sensations [34]. The MAIA includes multiple subscales assessing different aspects of interoception, such as awareness of bodily sensations, emotional awareness, and the ability to regulate emotions based on bodily signals. A higher score reflects greater interoceptive awareness. All the subscales demonstrated good internal reliability in this study, with Cronbach’s alphas > 0.70.

The BI-AAQ assesses the degree to which individuals accept and take action in alignment with their values, regardless of their body image concerns [20]. The questionnaire evaluates psychological flexibility related to body image and is designed to measure acceptance of body-related thoughts and feelings. Higher scores indicate greater acceptance and flexibility regarding body image. The internal reliability in this study was good (Cronbach’s alpha = 0.79).

### Pilot group therapy protocol

The pilot intervention consisted of six weekly 90-minute group sessions divided into two modules, each comprising three sessions. Each module included one theoretical session followed by two sessions focused on guided exposure.

In Module 1, the first session collaboratively defined body image and its components: cognitive (negative thoughts and beliefs about the body), affective (feelings and emotions toward the body), behavioral (repetitive behaviors like body checking, e.g., mirror checking, weighing), avoidance strategies (e.g., avoiding mirrors or wearing loose clothing), and perceptual (sensory modes of perceiving the body, including proprioception and interoception).

The behavioral component was emphasized, as body-checking and avoidance behaviors were identified as central mechanisms contributing to the maintenance of eating disorders. Participants explored their personal engagement with these behaviors and began identifying patterns.

Module 2 introduced the cognitive aspects of body image, focusing on judgmental internal dialogue, particularly the “inner critic.” Participants were guided to personify and name their inner critic, fostering psychological distancing and reducing its influence on body-related judgments.

The second and third sessions of each module involved individualized mirror exposure exercises, conducted in a group setting. Each participant engaged in a gradual, guided interaction with their reflection, while the other group members were present but seated away from the mirror area. This approach provided a supportive environment while maintaining individual focus.

The window of tolerance concept was integral to the intervention, serving as a tool to help participants manage emotional and physiological arousal during exposure exercises [35–37]. This concept, rooted in trauma-informed care, refers to the optimal range of emotional arousal within which individuals can function effectively. Within the window of tolerance, participants are able to engage with distressing stimuli, process emotions, and reflect adaptively. Outside the window of tolerance, participants might present hyperarousal (overactivation of the nervous system, leading to anxiety, emotional flooding, or panic) or hypoarousal (emotional numbing or dissociation, where individuals feel detached or “frozen”). Individuals with eating disorders often exhibit narrowed windows of tolerance, which predispose them to dysregulation in response to body-related stimuli. This dysregulation may result in avoidance behaviors or rigid coping mechanisms, such as excessive body checking or food-related rituals.

During exposure exercises, therapists used strategies to ensure participants remained within their window of tolerance, such as: grounding techniques (incorporating tactile elements or interactive group activities to anchor participants in the present moment), mindfulness practices (nonjudgmental awareness of bodily sensations and emotions), gradual exposure (allowing participants to engage incrementally with their reflections), and environmental adaptations (providing breaks or covering specific parts of the mirror).

After each exposure exercise, participants shared their experiences within the group. This debriefing emphasized normalizing emotional reactions and promoting a sense of shared understanding. Reflections often included discussions of initial discomfort, moments of insight, and gradual progress toward reducing avoidance and self-criticism.

The window of tolerance framework was also explicitly discussed, helping participants recognize their physiological and emotional states during exercises. For example, participants were encouraged to identify signs of hyper- or hypoarousal and implement regulation strategies, such as grounding, breathing techniques, or seeking support from the group. This self-awareness empowered participants to approach their distress with greater confidence and adaptability.

By integrating the window of tolerance with mindfulness, exposure, and group support, this intervention aimed to promote both body awareness and acceptance while reducing the rigidity of maladaptive body image patterns.

### Parallel treatments and potential confounds

All participants in the ED group received specialized inpatient treatment, which included nutritional rehabilitation, individual psychotherapy (cognitive-behavioral therapy), and medical monitoring (see Todisco et al [38] for further details). While all participants in the intervention group were undergoing standard treatment, the exposure-based intervention was provided as an additional component, and participants were not involved in any other structured body image therapy during the study period. However, given the multifaceted nature of ED treatment, improvements in body image flexibility may have been influenced by concurrent therapeutic interventions.

### Statistical plan

In our statistical analysis, we began by conducting a two-tailed t-test to assess potential significant differences in demographic and clinical variables between diagnostic groups, allowing for unbiased examination of group differences. We then performed a network analysis using JASP with the qgraph package, following the methodology outlined by the literature [39, 40]. In this analysis, we constructed a partial correlation network in which nodes represented measured variables, and edges signified conditional dependencies between variable pairs after controlling for all other variables in the dataset. Edge weights, depicted by line thickness, reflected partial correlation coefficients, while the absence of an edge indicated conditional independence between two variables. To minimize spurious connections, we applied the Least Absolute Shrinkage and Selection Operator (LASSO) regularization, which reduces smaller partial correlations to zero, and set the Extended Bayesian Information Criterion (EBIC) penalty parameter to 0.5, as recommended to control for sparse correlations [41]. Additionally, we calculated centrality measures, including strength, which reflects the sum of absolute edge weights connected to a node, to evaluate the importance of nodes within the network. To ensure accuracy, we performed nonparametric bootstrapping (*n* = 2500) to estimate confidence intervals on edge weights and assessed centrality stability by re-estimating the network and recalculating centrality indices after dropping incremental proportions of cases. The correlation stability (CS) coefficient was computed to determine the maximum proportion of cases that could be removed while maintaining a correlation of at least 0.7 with the original indices, with a threshold of 0.25 or higher deemed acceptable based on the literature recommendations [39]. Finally, to evaluate the impact of a pilot intervention on body image outcomes, we conducted a paired samples t-test, comparing pre- and post-intervention measures within the same participants.

## Results

### Group comparison

No significant differences were observed between groups in terms of age and BMI. However, as expected, notable differences emerged in body image concerns and specific subscales of the MAIA questionnaire. Body image evaluations indicated higher scores in the ED group, reflecting greater concerns. In contrast, the GP group showed higher scores on embodiment-related features in the MAIA, such as not-worrying, self-regulation, and trusting. See Table 1 for details.

**Table 1:** Demographic and clinical characteristics of the sample

	ED *n* = 69	GP *n* = 103	t	*p*
Age, years	24.43 8.47	23.66 2.17	0.743	0.460
BMI, kg/m^2^	19.24 8.53	21.26 2.83	-1.895	0.062
BUT				
Weight Phobia	3.35 1.13	1.90 1.42	7.420	< 0.001
Body Image Concerns	3.40 1.65	1.90 1.42	9.709	< 0.001
Avoidance	1.05 0.75	0.71 1.08	2.440	0.016
Compulsive Self-Monitoring	2.60 1.34	1.23 1.03	7.254	< 0.001
Detachment	1.90 0.84	0.81 1.10	6.965	< 0.001
BI-AAQ	33.29 13.12	60.20 21.27	-10.480	< 0.001
MAIA				
Noticing	2.74 1.03	2.43 1.32	1.725	0.086
Not Distracting	2.75 0.84	2.87 1.10	-0.786	0.433
Not Worrying	1.97 0.72	2.20 0.62	-2.237	0.027
Attention Regulation	1.71 0.98	2.06 1.00	-2.239	0.024
Emotional Awareness	2.53 1.15	2.64 1.38	-0.570	0.570
Self-Regulation	1.22 1.03	1.76 1.18	-3.064	0.003
Body Listening	1.20 0.99	2.01 1.24	-4.569	< 0.001
Trusting	0.84 1.09	2.70 1.41	-9.732	< 0.001

The table reports means and standard deviations. ED: eating disorder; GP: general population; BMI: body mass index; BUT: body uneasiness test; BI-AAQ: Body Image Acceptance and Action Questionnaire; MAIA: Multidimensional Assessment of Interoceptive Awareness

The ED group consisted of 49 women with anorexia nervosa (mean age 23.76 ± 8.27) and 20 women with bulimia nervosa (mean age 26.10 ± 8.94), with no significant differences in age between the subgroups (t = 1.04, *p* = 0.300). The duration of the disorders was also similar between the groups: anorexia nervosa had a mean duration of 5.73 ± 5.72 years, and bulimia nervosa had a mean duration of 7.30 ± 4.90 years (t = 1.07, *p* = 0.288).

### Network analysis findings

**Fig. 1:**
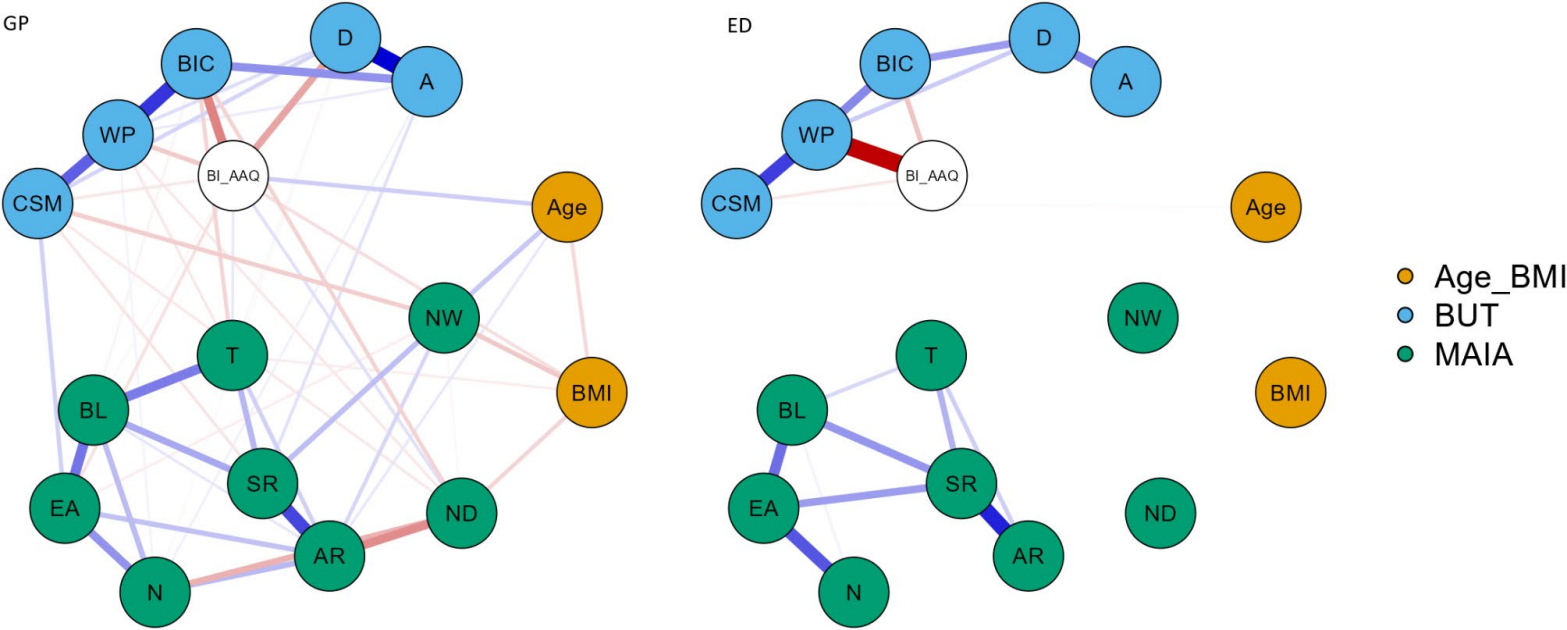
This network visualization compares two groups: individuals with eating disorders (ED) and a general population (GP) sample. Nodes represent variables, including the *Multidimensional Assessment of Interoceptive Awareness* (MAIA), the *Body Image-Acceptance and Action Questionnaire* (BI-AAQ), age, and BMI. Edges show the partial correlations between these variables, with blue edges indicating positive correlations and orange edges indicating negative correlations. The thickness of the edges reflects the strength of these relationships. The ED network (right) exhibits a fragmented structure, with limited connections and very few bridges between domains. The relationships are primarily contained within individual questionnaires (e.g., between MAIA subscales or BI-AAQ subscales), suggesting a disconnection between broader interoceptive awareness and body image flexibility. In contrast, the GP network (left) displays a more cohesive structure, with stronger connections across different variables, indicating a more integrated relationship between interoceptive awareness, body image flexibility, and demographic factors such as age and BMI. This network comparison illustrates the disrupted interconnections in the ED group, where body image and interoceptive awareness are less integrated, while the GP group shows a more connected and adaptive network. CSM: compulsive self-monitoring; WP: weight phobia; BIC: body image concerns; D: detachment; A: avoidance; N: noticing; ND: not distracting; NW: not worrying; AR: attention regulation; EA: emotional awareness; SF: self-regulation; BL: body listening; T: trusting; BMI: body mass index; BUT: body uneasiness test; BI-AAQ: Body Image Acceptance and Action Questionnaire; MAIA: Multidimensional Assessment of Interoceptive Awareness

**Fig. 2:**
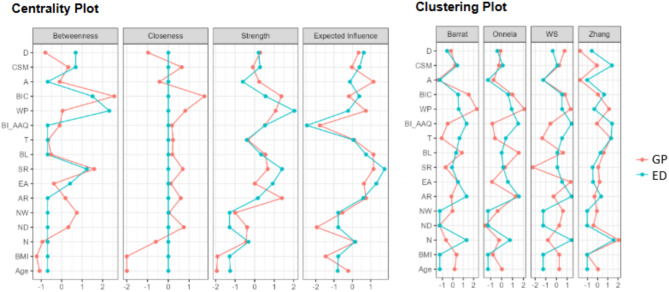
This figure presents the centrality and clustering indices for the networks of the ED and GP groups. Centrality measures the importance of nodes within the network, indicating how strongly each variable connects with others. High centrality scores suggest that a variable plays a central role in the network, while low centrality indicates weaker connections. The clustering indices represent the degree to which nodes in the network tend to cluster together, forming cohesive groups of closely related variables. A higher clustering coefficient indicates that variables in the network tend to form distinct clusters, while lower clustering values suggest more dispersed connections. The clustering indices in this figure further illustrate the more fragmented and isolated nature of the ED network compared to the more interconnected and adaptive structure observed in the GP group

**Fig. 3:**
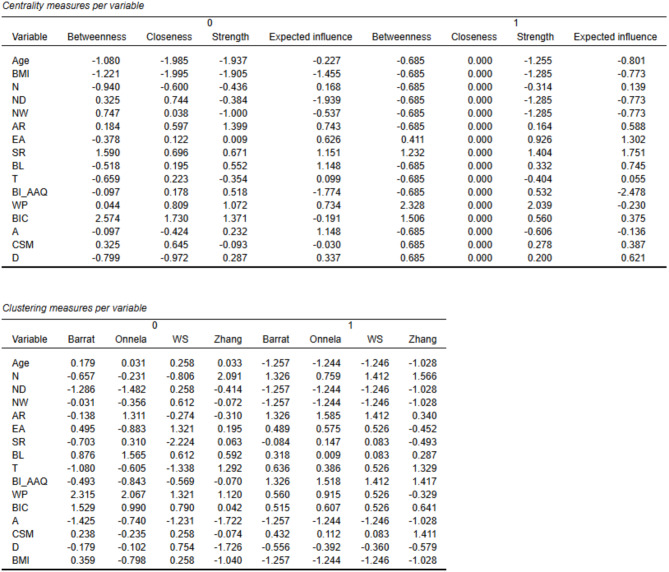
The tables present the data for the general population (GP, 0) and eating disorder (ED, 1) groups as shown in Fig. 2. CSM: compulsive self-monitoring; WP: weight phobia; BIC: body image concerns; D: detachment; A: avoidance; N: noticing; ND: not distracting; NW: not worrying; AR: attention regulation; EA: emotional awareness; SF: self-regulation; BL: body listening; T: trusting; BMI: body mass index; BUT: body uneasiness test; BI-AAQ: Body Image Acceptance and Action Questionnaire; MAIA: Multidimensional Assessment of Interoceptive Awareness

The network visualization is presented in Fig. 1, where blue edges represent positive partial correlations and orange edges indicate negative correlations. Figures 2 and 3 display the centrality and clustering plots for the variables within the networks. The correlation stability coefficients for both networks are above the threshold: 0.58 (GP) and 0.36 (ED).

In the GP network, the nodes with the highest strength centrality are attention regulation (1.00), body image concerns (0.99), weight phobia (0.93), and BI-AAQ (0.82). The strongest connections for attention regulation are with self-regulation (0.42), not-distracting (-0.26), and noticing (0.16). For body image concerns, the strongest connections are with weight phobia (0.46), BI-AAQ (0.28), and avoidance (0.25). The strongest connections for weight phobia are with compulsive self-monitoring (0.37) and BI-AAQ (-0.12).

In the ED network, the nodes with the highest strength centrality are weight phobia (1.00), self-regulation (0.81), emotional awareness (0.67), and body image concerns (0.56). Weight phobia shows its strongest connections with BI-AAQ (-0.36), body image concerns (0.17), and compulsive self-monitoring (0.27). The strongest connections for self-regulation are with attention regulation (0.31), body listening (0.15), and emotional awareness (0.14). Emotional awareness has its strongest connections with noticing (0.24), self-regulation (0.14), and body listening (0.20). For body image concerns, the strongest connections are with derealization (0.14), weight phobia (0.17), and BI-AAQ (-0.09). Closeness centrality across the ED network indicates a disconnected network structure.

### Pilot intervention outcomes

In the pilot evaluation, 24 women participated in the group protocol. The mean age was 19.79 ± 4.15 years, and the average BMI was 19.38 ± 5.35. The group consisted of 16 women with anorexia nervosa (mean age 19.00 ± 3.31) and 8 women with bulimia nervosa (mean age 21.38 ± 5.37), with no significant differences in age between the subgroups (t = 1.345, *p* = 0.145). The duration of the disorders was also similar between the groups: anorexia nervosa had a mean duration of 2.81 ± 3.31 years, and bulimia nervosa had a mean duration of 4.75 ± 3.01 years (t = 1.639, *p* = 0.115). The BMI differed significantly between the subgroups, with a mean of 16.43 ± 1.47 in the anorexia nervosa group and 23.40 ± 6.71 in the bulimia nervosa group (t = 2.91, *p* = 0.022).

Specific differences observed at the end of the group treatment included improvements in body trust as measured by the MAIA, body image flexibility, and reductions in weight phobia, self-monitoring, and body image concerns. See Table 2 for details.

**Table 2:** Effectiveness of pilot group therapy: before (T0) vs. After (T1)

	T0	T1	t (*p*)
BMI, kg/m^2^	19.38 5.35	21.01 3.84	-4.541 < 0.001
AN	16.43 1.47	19.81 2.28	-6.627 < 0.001
BN	23.40 6.70	23.42 5.23	-0.036 0.973
BUT			
Weight Phobia	3.72 0.99	3.13 1.05	3.203 0.004
Body Image Concerns	3.52 1.26	2.98 3.05	3.286 0.003
Avoidance	1.22 0.72	1.19 1.01	0.140 0.890
Compulsive Self-Monitoring	2.99 1.31	2.63 1.23	2.251 0.034
Detachment	2.09 0.82	1.90 0.81	1.113 0.277
BI-AAQ	31.75 12.13	38.29 12.60	-3.537 0.002
MAIA			
Noticing	2.79 1.21	2.81 1.01	-0.083 0.935
Not Distracting	2.78 0.93	2.68 0.81	0.455 0.653
Not Worrying	2.04 0.69	1.99 0.68	0.282 0.781
Attention Regulation	1.73 1.02	1.59 0.81	0.810 0.426
Emotional Awareness	2.55 1.22	2.75 1.02	-1.177 0.251
Self-Regulation	1.29 1.09	1.24 1.14	0.375 0.711
Body Listening	1.10 0.94	1.38 1.06	-1.624 0.118
Trusting	0.76 0.73	1.22 1.31	-2.210 0.037

BMI: body mass index; AN: anorexia nervosa; BN: bulimia nervosa; BUT: body uneasiness test; BI-AAQ: Body Image Acceptance and Action Questionnaire; MAIA: Multidimensional Assessment of Interoceptive Awareness

### Participants feedback of the pilot group

The pilot group intervention on body image received a range of feedback from participants, reflecting both the challenges and benefits of the approach. The group utilized gradual exposure through mindfulness to emotions, perceptions, sensations, thoughts, and behaviors related to the body and body image. This method counteracted avoidance and dissociation, common dysfunctional coping strategies in eating disorders that perpetuate distress and disconnection from the body. Many participants experienced strong ambivalence, describing a mixture of curiosity and desire for change alongside fear, anger, and disgust. One participant expressed this dynamic vividly: *“When I saw I could join the group*,* I was so happy and eager to accept and understand myself better. But during the sessions*,* I struggle to talk*,* feel frozen*,* and keep watching the clock*,* wanting to leave”.*

In the early sessions, the group focused on increasing awareness of maintaining mechanisms such as body checking, avoidance, and dysfunctional schemas. Participants often expressed surprise at the pervasiveness of these behaviors and their ambivalence about changing them. For example, one participant reflected: *“Yesterday*,* I tried counting how often I touch my stomach… I do it almost constantly! Now I realize how much of my day this consumes”.* While many recognized body checking as distressing and maintaining negative emotions, they also found challenging these behaviors daunting. Another participant noted: *“Even though I know looking in the mirror makes me feel worse*,* I can’t resist*,* especially after meals”.*

Small, manageable changes were reassuring to participants, and some reported significant benefits from addressing behaviors they had perceived as part of their identity. For instance, a participant shared: *“I threw away the jeans I used for three years to reassure myself I wasn’t changing*,* along with clothes I hadn’t worn in years. Now I feel a bit like my closet—empty*,* but also freer”.* Activities that introduced creative and sometimes playful elements, such as naming and personifying the internal critic, were generally well-received, though they occasionally brought up personal or traumatic memories. One participant described their internal critic as: *“A lion—majestic and reassuring*,* but also dominant*,* suffocating*,* and aggressive”.* Often, these reflections became valuable material for individual therapy sessions.

The second phase of each module involved more intense exposure, such as mirror work or mindfulness-based group activities. This phase was described as emotionally intense, intimate, and difficult but also transformative. Participants frequently noted that their immediate reactions involved self-judgment, characterized by rigid, critical, and objectifying thoughts: *“I immediately saw that the tops of my thighs touch”; “Messy*,* fat*,* short*,* ugly*,* insignificant”; “I couldn’t take my eyes off my arms—I wanted to dismantle them and reshape them”.* While some reported strong or absent emotional responses, others described increased emotional tolerance and a gradual reconnection with their bodily self. One participant reflected: *“At first*,* I felt anger toward myself*,* then disgust. But now*,* talking to the group*,* I feel sadness. It’s hard*,* but I notice I’m here and able to talk about it”.*

To mitigate the intensity of these experiences, individualized adaptations and group cooperation were essential. Techniques like taking breaks, playing with a ball, or covering parts of the mirror helped participants regulate overwhelming emotions. For instance, a participant shared: *“I started feeling light-headed and disconnected*,* but when a group member tossed me the ball*,* it brought me back. Gradually*,* I realized I wasn’t alone*,* and we ended the session hugging each other”.* Such adaptations supported participants in engaging with the exposure at their own pace, fostering collaboration and reducing judgment within the group.

Overall, participants noted several positive outcomes, including greater body awareness, reduced anxiety, and a less judgmental view of their physical selves. Gradual mirror exposure helped many gain a more realistic perception of their body, challenge distorted views, and reintegrate their body image. Some participants used these sessions as opportunities to revisit memories, occasionally traumatic ones, which they brought to individual therapy for further exploration. However, a few participants, especially those with severe symptoms or those introduced to the group prematurely, reported amplified focus on perceived flaws and heightened distress, highlighting the importance of careful timing and readiness for this intervention.

## Discussion

This study aimed to explore differences in body image flexibility and embodiment between individuals with EDs and a GP sample, while also conducting a pilot evaluation of a body image intervention in individuals with EDs. By integrating an exploratory analysis of the interconnections between these constructs with a preliminary assessment of a targeted intervention, this study provides both foundational insights into body image flexibility and embodiment in clinical and non-clinical populations and initial evidence on potential therapeutic applications. Our findings reveal significant variations in the manifestation of body image flexibility and interoceptive awareness across these groups, while the pilot intervention suggests that targeted approaches may enhance these constructs in individuals with EDs. This integrated approach allows for both a theoretical investigation of body image-related mechanisms and an initial evaluation of treatment strategies, paving the way for future research.The GP group exhibited higher scores on several dimensions of interoceptive awareness, including self-regulation, not-worrying, and trust. These higher scores suggest that individuals without EDs are more attuned to their internal bodily sensations and can regulate emotions effectively through this awareness. In contrast, the ED group displayed significantly higher scores on body image concerns and weight-related distress, reflecting the prominence of body image disturbances in this population. These findings align with existing literature, which highlights the difficulties individuals with EDs experience in embodied perception, often with a pronounced focus on body image concerns even after years of disorder [42, 43].

In the network analysis, we observed a cohesive structure in the GP group, where interoceptive awareness, body image flexibility, and body-related concerns formed an interconnected network. Specifically, attention regulation, body image concerns, and weight phobia were central nodes, with strong links between regulatory processes and body-related evaluations. This structure suggests that for individuals in the GP group, body image acceptance and flexibility extend beyond body weight and appearance to encompass a broader, adaptive framework of self-regulation and emotional awareness. This adaptive framework fosters a more resilient and less appearance-driven relationship with one’s body, supporting findings that emotional regulation, mindfulness, and self-compassion contribute to healthier body image perceptions and improved mental well-being [44–46]. Such approaches emphasize the importance of psychological flexibility in navigating body-related distress, promoting a holistic and sustainable acceptance of the body.

Conversely, the ED network was marked by a more fragmented structure. Weight phobia emerged as the most central node, with strong connections to body image concerns and compulsive self-monitoring but fewer connections to broader regulatory or emotional processes. This pattern indicates that weight and appearance concerns may drive the psychological experience in EDs, anchoring body-related acceptance solely within symptomatic, appearance-based concerns rather than a broader, resilient sense of self-acceptance [47–50]. This network fragmentation suggests difficulty in integrating emotional and regulatory processes, which may reinforce the rigidity and distress often seen in ED-related body image disturbances.

### Pilot group therapy intervention

In the pilot evaluation of the targeted group therapy intervention, the ED group displayed improvements in key dimensions such as body trust, body image flexibility, and a reduction in weight phobia, self-monitoring, and body image concerns. These preliminary results suggest that targeted therapeutic interventions may help individuals with EDs develop a more resilient and adaptive body image by fostering internal body awareness and enhancing their capacity to accept body-related thoughts and feelings [51].

Specific improvements in body trust and interoceptive awareness might indicate that participants may benefit from therapy approaches that emphasize a re-engagement with bodily sensations, potentially strengthening their ability to navigate body-related distress without resorting to maladaptive behaviors. Notably, these changes imply that therapeutic efforts to reduce excessive focus on weight and appearance while building body acceptance and regulatory capacity could alleviate some of the most central issues in ED-related body image pathology.

Participant feedback from the pilot intervention provided valuable context for understanding the challenges and potential impacts of body image therapies. Feedback highlighted the significant ambivalence many participants faced when confronting body-related distress, as well as the transformative potential of exposure-based techniques. These insights emphasize the importance of individualized adaptations and gradual exposure in therapy, particularly for individuals with severe ED symptoms.

### Clinical implications

The findings suggest several potential clinical applications. First, enhancing the connection between interoceptive awareness and body image flexibility could provide a valuable resource for individuals with EDs, helping them utilize bodily awareness as a tool for managing body uneasiness. For instance, interventions that focus on strengthening bodily awareness and acceptance could enable individuals to internalize more adaptive body perceptions, which in turn may alleviate some of the psychological strain linked to body dissatisfaction and weight concerns [52, 53].

Second, in the ED group, targeting weight phobia and compulsive self-monitoring as core components of the treatment could facilitate a broader and more integrative approach to body image acceptance [54, 55]. Expanding the scope of BI-AAQ connections beyond weight and appearance-related concerns—potentially through therapy that focuses on self-compassion and emotional regulation—may foster a more comprehensive and unconditional acceptance of the body. Ultimately, such an approach could enhance resilience, reduce the centrality of weight-focused distress, and contribute to a more stable body image.

### Limitations

This study has several limitations. First, the cross-sectional design restricts our ability to draw causal inferences about the relationships between body image flexibility, interoceptive awareness, and body-related concerns. Longitudinal studies would be essential to determine whether changes in one domain (e.g., increased interoceptive awareness) lead to corresponding changes in body image flexibility and related constructs. Second, the sample consisted exclusively of female participants, limiting the generalizability of findings to male or non-binary populations who may experience body image differently. Further research involving diverse genders could help refine our understanding of these constructs across various groups. Third, while participant feedback offers valuable qualitative insights into the experiences of those undergoing the intervention, the self-reported nature of this data may be subject to bias. Moreover, the intensity of emotions described during the intervention highlights the importance of readiness and individualized pacing, suggesting that the findings may not generalize to all individuals with EDs.

Additionally, while the pilot intervention findings suggest potential therapeutic benefits, the small sample size and lack of a control group limit the strength of these conclusions. Future studies with larger, randomized samples could provide more definitive evidence on the efficacy of targeted group therapies for body image flexibility and embodiment in ED populations. Cultural and demographic factors were not fully explored, which may influence body image experiences and interoceptive awareness differently across populations. Finally, this study was not preregistered, which may introduce the risk of data-driven hypothesis generation and selective reporting. While our analyses were guided by theoretical considerations, future research would benefit from preregistration to enhance methodological transparency, reduce potential biases, and improve replicability.

## Conclusion

In conclusion, this study provides valuable insights into the differential structures of body image flexibility and embodiment in ED and GP samples. While the GP group demonstrates a cohesive and adaptive network where interoceptive awareness and body image flexibility are tightly linked to regulatory processes, the ED group reveals a more fragmented structure. In EDs, weight phobia and body image concerns predominate, with weaker links to broader regulatory and awareness processes, reflecting the rigidity often associated with disordered eating.

Participant feedback underscores the complexity and emotional intensity of body image interventions. By gradually addressing body image concerns and fostering collaboration and adaptability, therapeutic strategies can promote meaningful changes while mitigating distress.

The pilot intervention results underscore the potential for therapeutic approaches that enhance body trust, interoceptive awareness, and body image flexibility, thus reducing the intensity of weight-related concerns and self-monitoring behaviors. These findings emphasize the need for therapeutic interventions that focus on both the reduction of body-related distress and the promotion of a resilient, integrative body image acceptance, ultimately offering a pathway to improved psychological functioning for individuals with EDs. Future research should aim to replicate these findings in diverse populations and further explore the mechanisms underlying these complex constructs, contributing to evidence-based interventions tailored to ED-specific body image challenges.

## Data Availability

No datasets were generated or analysed during the current study.
